# Chemically stable fluorescent anti-counterfeiting labels achieved by UV-induced photolysis of nanocellulose†

**DOI:** 10.1039/d1ra02089g

**Published:** 2021-05-21

**Authors:** Hongrui Cheng, Xiaofeng Wei, Haijiang Qiu, Wensong Wang, Wenyue Su, Yuanhui Zheng

**Affiliations:** College of Chemistry, Fuzhou University Fuzhou 350116 China suweny@fzu.edu.cn; National Engineering Research Center of Chemical Fertilizer Catalyst Fuzhou University Fuzhou 350002 PR China; Fujian Science & Technology Innovation Laboratory for Optoelectronic Information 350116 China Yuanhui.Zheng@fzu.edu.cn

## Abstract

Carbon-based fluorescent security labels are effective methods to prevent counterfeiting. However, the properties of poor optical stability, complex and energy-consuming synthesis processes and weak bonding with substrates of carbon-based fluorescent materials limit their application prospects. Here, a novel *in situ* fluorescent patterning strategy is developed to achieve covert, chemically stable and solvent-tolerant cellulose-based security labels by UV exposure. The unsaturated double bonds as the origin of the fluorescence were generated during the photodegradation process under UV exposure. The fluorescent emission of cellulose-based materials reveals excellent stability under acidic, alkaline, reducing, oxidizing and non-polar solvent environments. These advantages give the cellulose nanofiber based security label fantastic potential applications.

## Abstract 2

The cellulose-based fluorescent patterning strategy is developed by UV exposure to achieve covert, chemically stable and solvent tolerant security labels.

## Graphical abstract

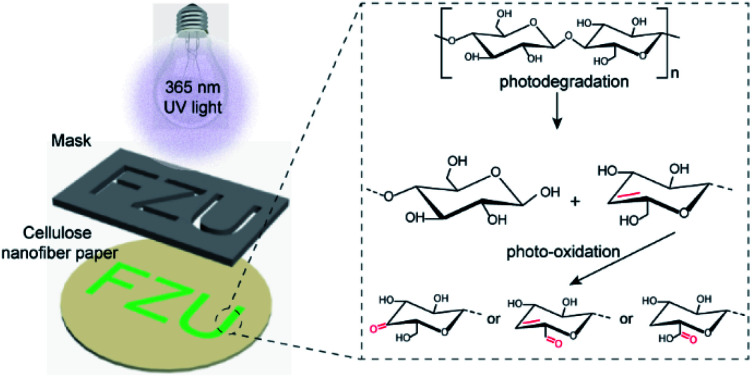

## Introduction

Counterfeiting has been a severe problem over the last few decades in scope and magnitude. Government and business organizations are concerned because of the adverse impact of such illicit activities. Counterfeiting also poses threats to the welfare of consumers, along with disrupting the whole of society on various levels.^1,2^ According to the Global Brand Counterfeiting Report 2018, the economic impact of global counterfeiting reached 1.2 Trillion USD in 2017.^3^ Therefore, it is urgent to develop innovative anti-counterfeiting materials and patterning technologies to combat counterfeiting. In this respect, fluorescent materials are regarded as ideal anti-counterfeiting materials owing to their robust, stable and tunable emission outputs in response to light that can be easily verified by eyes.^4^ So far, the widely investigated fluorescent materials involve organic dyes,^5,6^ inorganic semiconductor nanoparticles,^7^ II–IV group quantum dots,^4^ perovskite quantum dots,^8–10^ rare earth luminescent materials,^11,12^ and carbon dots (CDs).^13,14^ Among these materials, carbon-based fluorescent materials have attracted great interest for the next generation of anti-counterfeiting labels, owing to their high abundance and high degree of structural designability.

Organic dyes and CDs are two common carbon-based fluorescent materials. The former requires multi-step chemical synthesis and suffers from poor thermostability, easy photobleaching and fluorescence quenching,^15,16^ while the latter requires complicated separation steps for purification, such as multiple cycles of centrifugation or long-time dialysis.^17^ These materials are usually used as inks for security labels, in which the ink is embedded or coated on a print substrate, such as plastic films or papers. The inks that are integrated with the substrates *via* hydrogen bonding and physical adsorption could be easily removed by solvent, depending on the solubility of the ink in the solvent.^18,19^ The information displayed by fluorescent inks is at risk of being erased. To solve the issues, it is necessary to design a new strategy of fluorescent patterning technique that must satisfy the following criteria: (i) the print substrate itself can act as ink materials (*i.e.*, information carrier); (ii) it has to be environment-friendly, cheap and easy to obtain; (iii) it has to possess excellent chemical stability under various conditions, *e.g.*, under acidic, alkaline, reducing and oxidizing environments. Thus, developing low-cost and straightforward strategies for the facile production of carbon-based fluorescent materials is highly desired.

Cellulose, one of the most abundant, biodegradable and non-toxic natural polymers, is an ideal candidate that meets the above requirements.^20,21^ Cellulose is formed by condensing through β (1 → 4)-glycosidic bonds.^22^ It has been previously reported that the photodegradation and photo-oxidation of cellulose can undergo UV light irradiation, which is due to a radical-based autooxidation process in the absence of catalysts.^23,24^ The glycosidic bonds of cellulose are cleaved to randomly form unsaturated bonds that act as capture centers of excitons and give rise to fluorescence,^25^ offering the material the optical information storage ability.^26^ Also, the hydroxyl groups on the glucose from one chain form hydrogen bonds with oxygen atoms of the hydroxyl groups on the same or on a neighbor chain, holding the chains firmly together side-by-side and forming microfibrils with high tensile strength.^27^ According to these properties, cellulose can be developed to fluorescent security labels through photolithography.

In this work, we developed a stable fluorescent security label using environmentally friendly and renewable cellulose nanofibers as both ink and print substrate through photolithography. During the photo-oxidative degradation of cellulose, the unsaturated double bonds forming on the glucose units were the origin of the fluorescence property. The *in situ* formed fluorescent patterns on cellulose nanofiber papers is covert, solvent resistible and chemically stable. The fluorescence emission peak of cellulose nanofiber paper was tunable from 440 nm to 490 nm by adjusting the photo-oxidation time. It is important that the fluorescence information is stable in various extreme chemical environments (*e.g.*, acidity, alkalinity, reducibility, oxidability, and non-polar solvent environments). The cellulose-based security labels have shown a great potential application in the anti-counterfeiting field.

## Results and discussion

**Fig. 1:**
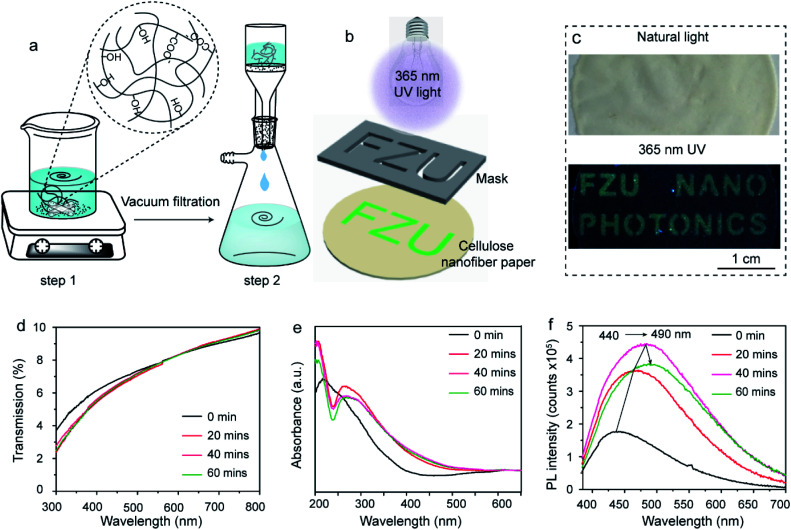
Schematic depicting the fabrication of (a) cellulose nanofiber paper and (b) 2D fluorescence patterns, (c) photographs of the cellulose nanofiber paper based security label under natural light (top) and under 365 nm UV light (bottom), and (d) transmission, (e) absorption and (f) fluorescence spectra of a cellulose nanofiber paper exposed to 365 nm UV light for different time.

The fabrication of cellulose nanofiber paper is schematically illustrated in Fig. 1a. The building blocks (*i.e.*, cellulose nanofibers) of the paper were extracted from garlic husk.^27–29^ It is well known that natural garlic husk consists of cellulose, hemicellulose, lignin and ash. The hemicellulose and lignin residues were removed during the NaOH/NaClO treatment and H_2_SO_4_ treatment, respectively.^27,30^ The cellulose nanofiber suspension was uniformly dispersed after high-speed stirring (step 1). Subsequently, the mixture was subjected to filtration under vacuum, forming a robust, flexible and transparent paper (step 2). During the vacuum filtration process, the cellulose nanofibers were stacked together *via* hydrogen bonds, which gives the paper excellent mechanical property.^29^ The cellulose nanofiber paper with a thickness of 20 μm can withstand the stress of 10 N (about 1 Kg) without breaking (Fig. S1 ESI†). The tensile strength and Young's modulus of the paper are 43.5 MPa and 450 ± 60 MPa, respectively (Fig. S1 ESI†). Fig. 1b schematically illustrates the fabrication of two-dimensional (2D) fluorescence patterns on the cellulose nanofiber paper. As shown in Fig. 1b, the fluorescent pattern was achieved by irradiating UV light from a portable UV lamp with a wavelength of 365 nm through a pre-defined photomask placed on the top of the fabricated paper. The fluorescence quantum yield of the irradiated area is measured to be 1.5% at 365 nm excitation.

Any 2D features, such as letters, pictures and patterns, can be generated. Fig. 1c shows the photographs of a cellulose nanofiber paper-based security label. Letters of “FZU NANO PHOTONICS” were written on the cellulose nanofiber paper. Despite of the relative low fluorescence quantum yield of the patterned area, the letters can still be easily seen by naked eyes under 365 nm UV light excitation as they show green color (bottom panel of Fig. 1c) but not in natural light (top panel of Fig. 1c). Fig. 1d–f show the evolution of the transmission, absorption and fluorescence spectra of the cellulose nanofiber paper after exposure to UV light for different time. After UV exposure, there is only a small difference in transmittance of the paper in the region of visible range from 300 nm to 800 nm (Fig. 1d), which explains the covert character of the generated 2D patterns discussed above. However, its absorption profile changed significantly in the 200–400 nm region (Fig. 1e). More specifically, the broad absorption peak of the paper splits into two peaks and the absorption edge red-shifts after the UV exposure, which indicates to the change of the structure or the formation of a new compound. It should be noted that the change of absorbance (absorption intensity) is very small between the investigated samples, in consistent with the transmittance of the samples. According to the literature, the increasing absorbance at 260 nm is caused by the formation of unsaturated double bonds, alkenyl, aldehydes and ketones.^31^Fig. 1e shows the fluorescence emission spectra of the pristine and UV exposed cellulose nanofiber papers under 365 nm excitation. As expected, the maximal emission peak red-shifts from 440 nm to 490 nm with prolonging UV exposure time. It is also found that the fluorescence intensity increases first and then decreases with the increase of UV irradiation time.

**Fig. 2:**
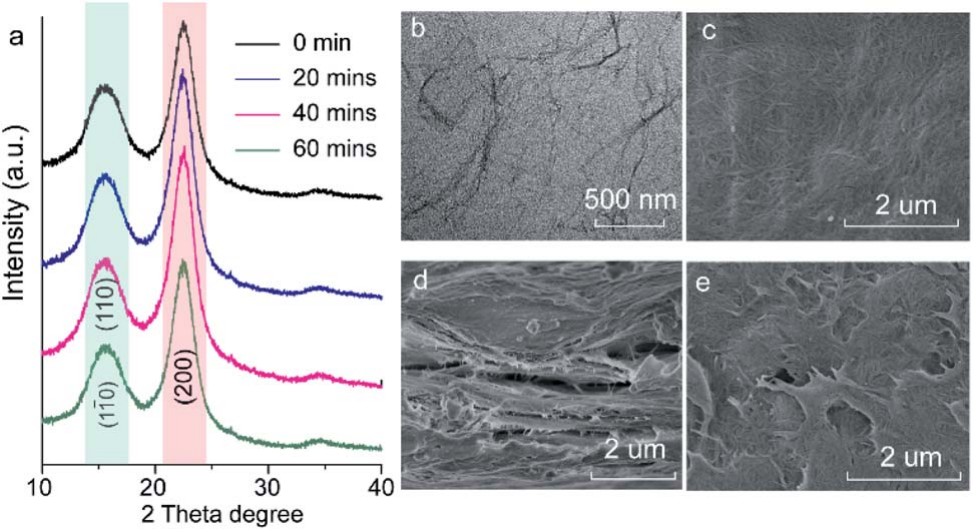
(a) XRD patterns of the fabricated cellulose nanofiber paper before and after different UV-irradiation time. (b) A typical TEM image of the as-prepared cellulose nanofibers, and (c–e) typical SEM images of the cellulose nanofiber paper. Note that panel c is the top view of the pristine cellulose nanofiber paper, panel d the cross-section view of the pristine paper, and panel e the top view of the paper after 40 min UV irradiation.

Fig. 2a shows the XRD patterns of the fabricated cellulose nanofiber paper before and after different UV-exposure time. Two strong and broad diffraction peaks at 15° and 22.5° assigned to the (110)/(11̄0) and (200) crystal facets of cellulose are observed for all the samples.^32^ There is no obvious shift of the cellulose diffraction peaks, indicating that the backbone of the cellulose remains unchanged upon UV irradiation. Fig. 2b shows a typical transmission electron microscopy (TEM) image of the prepared cellulose nanofibers. 1D nanofibers with a width of about 5 nm and a length ranging from a few hundred nanometers to several micrometers are observed. Some of the nanofibers stack together, forming larger fibers. The top-view of the pristine paper shows that the 1D cellulose nanofibers entangle together and form a connected 2D meshwork structure (Fig. 2c). Such lamellar structures further stack together layer-by-layer, as presented in Fig. 2d. One can easily be seen that the surface of the paper is very smooth prior to UV exposure (Fig. 2c). However, it becomes rough after the UV exposure (Fig. 2e). It should be noted that the 2D lamellar structures shrink after UV irradiation (Fig. 2e), indicating that the cellulose nanofibers underwent photodegradation during UV exposure process.

**Fig. 3:**
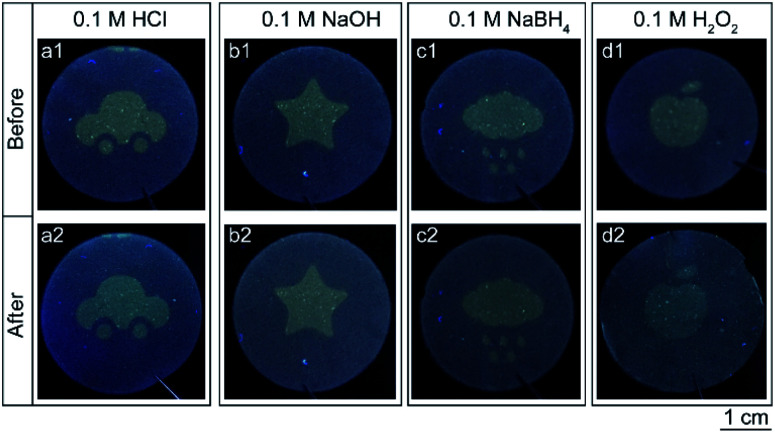
Photographs of the cellulose nanofiber paper based security labels in various solvents. The photographs in the first row and the second row were taken before and after the solvent treatment, respectively.

Ideal security labels have to be stable during their practical application. To investigate the pattern stability of the prepared security labels, they were subjected to various chemical environments, such as acidic, alkaline, reducing, oxidizing and non-polar solvent environments. Fig. 3 shows the photographs of the cellulose nanofiber paper based security labels before and after the exposure to HCl (0.1 M), NaOH (0.1 M), NaBH_4_ (0.1 M) and H_2_O_2_ (0.1 M) solutions for 6 hours. In some scenarios, the colors of the patterns changed slightly after the treatments (Fig. 3 columns a and d). Yet, for all the scenarios, the shapes of the patterns remain identical and can be clearly seen by eyes under UV light excitation. In addition, the fluorescent patterns of the cellulose nanofiber paper based security labels also keep unchanged when exposed to various non-polar solvents, such as petroleum ether and benzene (Fig. S2 ESI†). For comparison, security labels consisted of methylene blue or fluorescein were prepared and underwent *N*,*N*-dimethylformamide (DMF) treatment, NaBH_4_ treatment, and ethanol treatment respectively. The main reason for choosing methylene blue as the model dye to prepare the reference security label is that it can not only easily dissolve in an organic solvent but also be switched between blue color in an oxidizing state and colorless in a reducing state. The corresponding printing patterns (*i.e.*, the logo of Fuzhou University) were destroyed when exposed to the DMF solvent due to the dissolution of the dyes in the solvent or became invisible due to the reduction of the dyes (Fig. S3 ESI†). Likewise, the logo printed by fluorescein were destroyed when immersed in ethanol solvent (Fig. S3 ESI†). The above results reveal that, compared with conventional fluorescent security labels, the cellulose nanofiber paper based security labels exhibit much better chemical stability under acidic, alkaline, reducing, oxidizing and non-polar solvent environments.

**Fig. 4:**
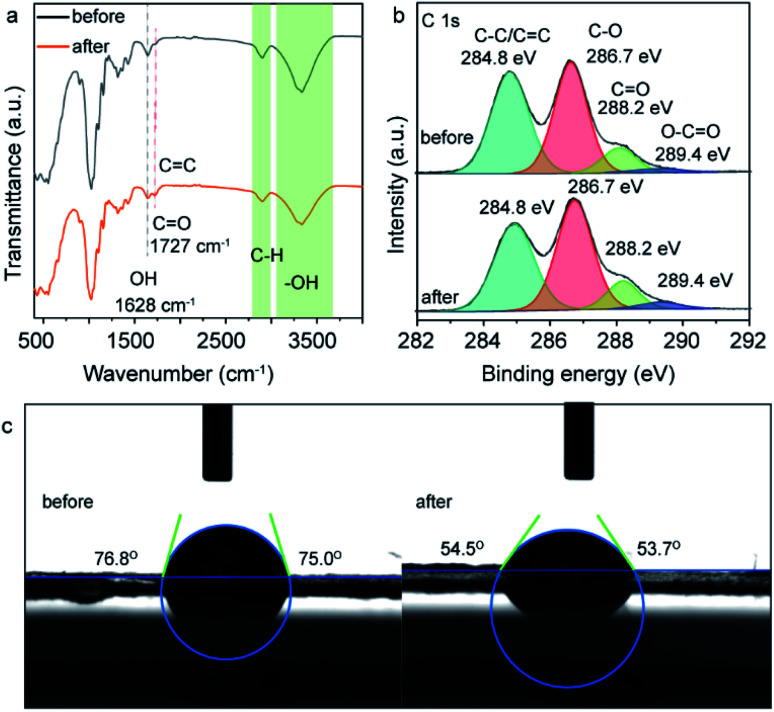
(a) FTIR spectra, (b) high-resolution C1s XPS spectra, (c) and photographs of contact angle of the cellulose nanofiber paper before and after UV light irradiation.

To understand the origin of fluorescence of the cellulose nanofiber paper after UV exposure, the samples were characterized by FTIR and XPS (Fig. 4). As recorded in Fig. 4a, the IR peaks at 896 cm^−1^ (C–H rocking vibration), 1025/1053 cm^−1^ (alkoxy C–O stretching vibration), 1103 cm^−1^ (glucose ring stretching vibration), 1159 cm^−1^ (C–O–C asymmetric stretching vibration), 1315 cm^−1^ (O–H in-plane deformation), 1366 cm^−1^ (C–H symmetric deformation), 1428 cm^−1^ (C–H asymmetric deformation), 1628 cm^−1^ (stretching vibration of adsorbed OH), 1680 cm^−1^ (C=C stretching vibration), 1727 cm^−1^ (C=O stretching vibration), 2897 cm^−1^ (C–H stretching vibration), and 3285–3330 cm^−1^ (O–H stretching vibration) are ascribed to the fingerprint peaks of cellulose, and the strong and wide IR peak in the range of 3000–3700 cm^−1^ belongs to hydrogen bonds formed between the cellulose nanofibers.^33,34^ The formation of hydroxyl bonds makes the cellulose nanofibers more tightly bound together within the paper. It should be noted that, upon UV light exposure, the peak intensity of hydroxyl groups decreases, while the peak intensity of C=O groups significantly enhances and shifts to lower wavenumber. This means that some hydroxyl groups were converted to C=O groups (probably carboxyl acid, aldehyde or ketone groups) and some alkenyl groups were formed. The high-resolution C 1s XPS spectra of the same samples shows that there are four different types of carbon species with binding energy at 284.8 eV, 286.7 eV, 288.2 eV and 289.4 eV, which are assigned to the carbon in C–C/C=C, C–O, C=O and O–C=O groups, respectively.^28,29^ Interestingly, when using C–C peaks as a reference, the intensity of the C 1s XPS peaks at 286.7 eV, 288.2 eV and 289.4 eV increase slightly after UV light exposure, indicating partial of hydroxyl groups were converted to C–O, C=O and O–C=O groups during the UV exposure. The increase of the amount of carboxyl acid groups on the area exposed to UV light can lead to the increased hydrophilia of the cellulose nanofiber paper, as carboxyl acid groups are more hydrophilic than hydroxyl groups. To prove this, we measured the contact angle of the cellulose nanofiber paper before and after UV light irradiation. The side-view of a droplet on the cellulose nanofiber papers is presented in Fig. 4c. The contact angle of the paper is 75° and 54° before and after UV light exposure. The decrease of the contact angle further confirms the partial conversion of carboxyl groups into carboxyl acid groups.^35^ According to the Raman spectrum of the UV irradiated cellulose nanofiber paper (Fig. S4 ESI†), no peaks of sp^2^ hybridized carbon are observed, indicating that there are not carbon dots have been generated.^36^

**Fig. 5:**
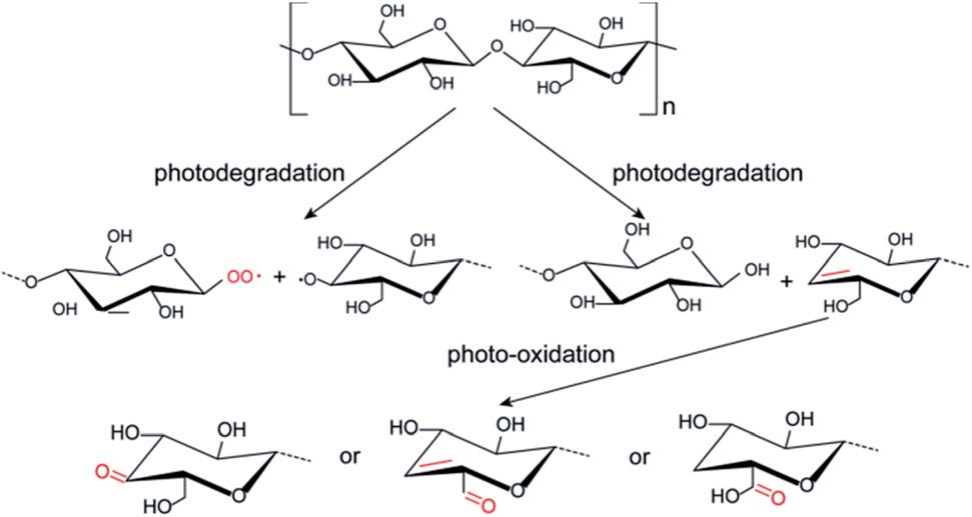
Schematic diagram of the photochemical reaction mechanism for the formation of fluorescent patterns on the cellulose nanofiber paper under UV light irradiation.

Based on the above results, the possible photochemical reaction mechanism for the formation of photo-induced fluorescent patterns on the cellulose nanofiber paper is proposed in Fig. 5. The glycosidic bonds between two neighboring glucose units undergo two different degradation reactions during the photolithography, forming a pair of –C–O˙ and C–O–O˙ radicals and a pair of a unsaturated double bond and a hydroxyl group on the glucose units, respectively.^24,31^ The formation of the peroxy radicals explains the increase of the C–O intensity in the C 1s XPS after UV exposure. It has been reported that the unsaturated double bonds can act as the recombination centers of excitons, which gives the materials the property of fluorescence.^26^ This causes the red-shift of the absorption and emission peaks.^37^ Further increasing the UV exposure time results in the oxidation of partial hydroxymethyl groups (–CH_2_–OH) into aldehyde groups and carboxyl acid groups,^23^ which are polar π-conjugated groups (*i.e.*, electron acceptor groups) and can draw electron from the formed unsaturated bond on neighbor carbon atom.^26^ Similarly, the unsaturated double bonds (C=C) can also be oxidized by the singlet oxygen during the further UV-induced photolysis progress, resulting in the irreversible quench of the fluorescence.^38^ This causes the decrease of the fluorescent intensity of the degraded cellulose, in consistent with the fluorescence results in Fig. 1f. For the area that is not exposed to UV light, no reactions occur on cellulose nanofibers, and thus their optical property remains unchanged. Therefore, a pre-defined pattern on the mask was transferred onto the cellulose nanofiber papers.

## Conclusions

In summary, we have demonstrated an inkless printed, covert, stable and fluorescent security label, which was fabricated by photolithography of self-assembled nanocellulose paper. The renewable cellulose nanofibers on the surface were photodegraded and photo-oxidized during the UV exposure and formed unsaturated double bonds that are responsible for the green fluorescent light emission under UV excitation. The information generated by UV light is covert and shows excellent chemical stability under acidic, alkaline, reducing and oxidizing environments. The increase of the crystallinity of the photolysized nanocellulose or the introduction of metal ion doping may achieve room temperature phosphorescence for optical encryption,^39,40^ further increasing the encoding level of the anti-counterfeiting labels. The cellulose nanofibers act as inks and substrates, which is a great strategy to reduce the steps and energy consumption required to prepare security labels. This work represents a simple method to write information on the surface of cellulose nanofiber paper, which could be used as security labels and information storage devices.

## Experimental

### Materials

Methylene blue (AR grade, from Aladdin Reagents Shanghai Co., Ltd.), fluorescein (AR grade, from Sigma-Aldrich Shanghai Co., Ltd.), hydrochloric acid (HCl, AR grade, from Zhejianglanxi Xuri Chemical Reagents Co., Ltd.), sulfuric acid (H_2_SO_4_, AR grade, from Zhejianglanxi Xuri Chemical Reagents Co., Ltd.), sodium hypochlorite solution (NaClO, AR grade, from Xilong Scientific Co., Ltd.), sodium hydroxide (NaOH, AR grade, from Xilong Scientific Co., Ltd.), sodium borohydride (NaBH_4_, AR grade), hydrogen peroxide (H_2_O_2_ 30% AR grade), were purchased from Sinopharm Chemical Reagents Co., Ltd. (Shanghai, China). All reagents mentioned above were used as received.

### The extraction of cellulose nanofibers from garlic husk

Extraction of cellulose nanofibers from garlic husk was conducted using a previously reported method with minor modification.^27–29^ In a typical procedure, 10 g of dried garlic husk was immersed in 400 g of NaOH solution (2 wt%) at 140 °C for 5 h and then washed with ultrapure water. Subsequently, the obtained garlic skin was added to a mixture of 200 g of H_2_SO_4_ solution (2 wt%) and 300 g of sodium hypochlorite (1.5 wt%) and stirred at 80 °C for 6 h to remove the lignin. The resulting cellulose slurry was ultrasonicated at 600 W for 1 h and then stirred at 1200 rpm for 1 h to obtain a cellulose nanofiber suspension, which was washed with ultrapure water through centrifugation three times to remove the chemical reactants and finally re-dispersed in ultrapure water.

### Preparation of cellulose-based fluorescent security labels

Cellulose nanofiber paper was prepared through the self-assembly assisted vacuum filtration method. In a typical procedure, 10 mL of cellulose nanofiber suspension (3.33 mg mL^−1^) were filtered to form a paper under vacuum. The fluorescent patterns were prepared through photolithography. More specifically, a pre-defined photomask was placed on the top of cellulose nanofiber paper. The UV light with wavelength of 365 nm was irradiated through the mask using a 6 W handheld UV lamp as the light source.

### Characterization

An X-ray diffractometer (XRD, RIGAKU Ultima IV) with Cu Kα radiation (*λ* = 0.15406 nm) was used to characterize the crystal phases of the fabricated cellulose nanofiber paper. A transmission electron microscope (TEM, Tecnai Model G2 F20 S-TWIN) was used to examine the morphology of the cellulose nanofibers. A high-resolution field emission scanning electron microscope (SEM, Verios G4) was used to characterize the morphology of the cellulose nanofiber paper. The surface elemental states of the cellulose nanofiber papers were analyzed by X-ray photoelectron spectroscopy (XPS, VG ESCALAB 250) with a monochromatized Al kα X-ray source (15 kV, 150 W, 500 nm, pass energy 20 eV). The binding energy of C1s (284.8 eV) was used for spectral calibration. The optical properties were evaluated by UV-vis spectrophotometer (UV-2600, Shimadzu Co.) and a fluorophotometer (Edinburgh FL/FS900). The Fourier transform infrared (FTIR) spectra were measured on an FTIR spectrometer (Nicolet-iS50, Thermo Scientific Co.).

## Author contributions

H. C. and Y. Z. conceived the idea. H. C. and X. W. designed experiments and synthesized material. H. Q. and S. W. done transmission and fluorescent spectra test. W. S. and Y. Z. was involved in programming and data and supervised the project. H. C., X. W. and Y. Z. wrote the manuscript and all authors reviewed it.

## Conflicts of interest

There are no conflicts to declare.

## Supplementary Material

RA-011-D1RA02089G-s001
