# Supplementary material for: Chemically stable fluorescent anti-counterfeiting labels achieved by UV-induced photolysis of nanocellulose
Source: RSC Adv. 2021 May 21;11(30):18381–6. doi: 10.1039/d1ra02089g (PMC9033396; doi:10.1039/d1ra02089g)
Supplement: RA-011-D1RA02089G-s001 [file RA-011-D1RA02089G-s001.pdf]

Electronic Supplementary Material (ESI) for RSC Advances.
This journal is © The Royal Society of Chemistry 2021

Supporting information

Chemically stable fluorescent anti-counterfeiting labels achieved by UV-induced photolysis of nanocellulose

Hongrui Cheng[a ‡], Xiaofeng Wei[b ‡], Haijiang Qiu[a], Wensong Wang[a], Wenyue Su[a] *, Yuanhui Zheng[a c] *

[a] College of Chemistry, Fuzhou University, Fuzhou 350116, China.

[b] National Engineering Research Center of Chemical Fertilizer Catalyst Fuzhou University Fuzhou, 350002, PR China

[c] Fujian Science & Technology Innovation Laboratory for Optoelectronic Information, 350116, China.

† These authors contributed equally to this work

* Corresponding author: suweny@fzu.edu.cn; Yuanhui.Zheng@fzu.edu.cn

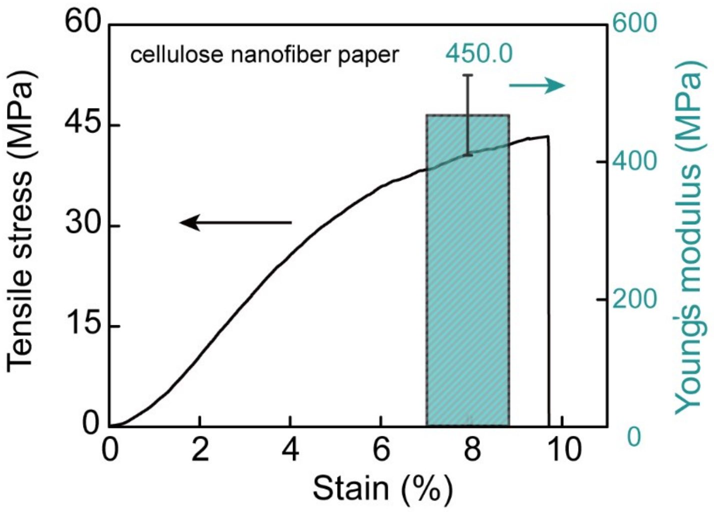

Fig. S1. Tensile stress-strain curve and Young's modulus of the cellulose nanofiber paper.

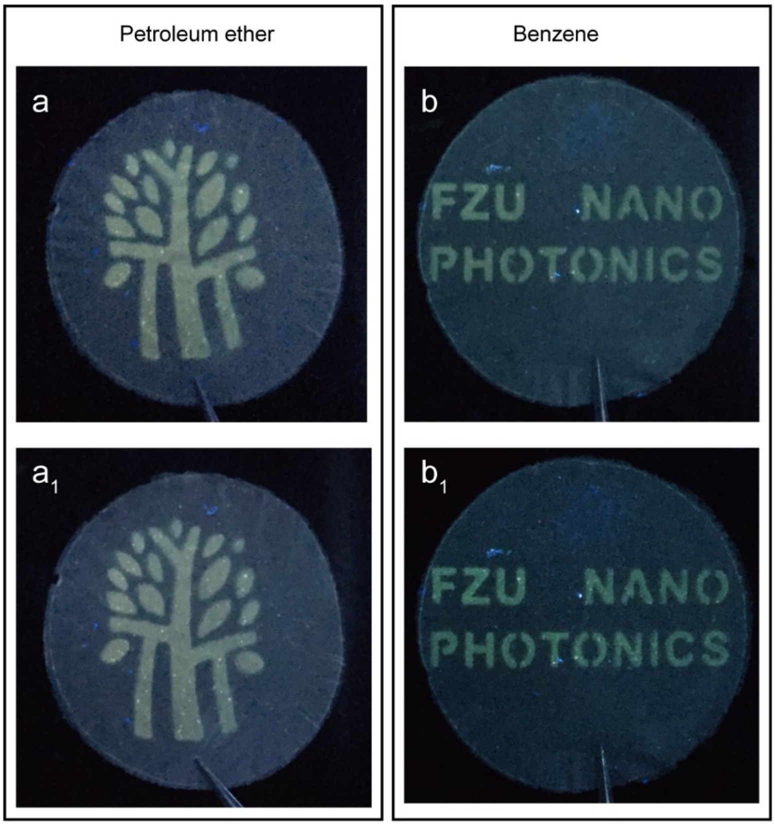

Fig. S2. The photography of cellulose nanofiber papers immersed in different organic solvents, before (the first row), after (the second row).

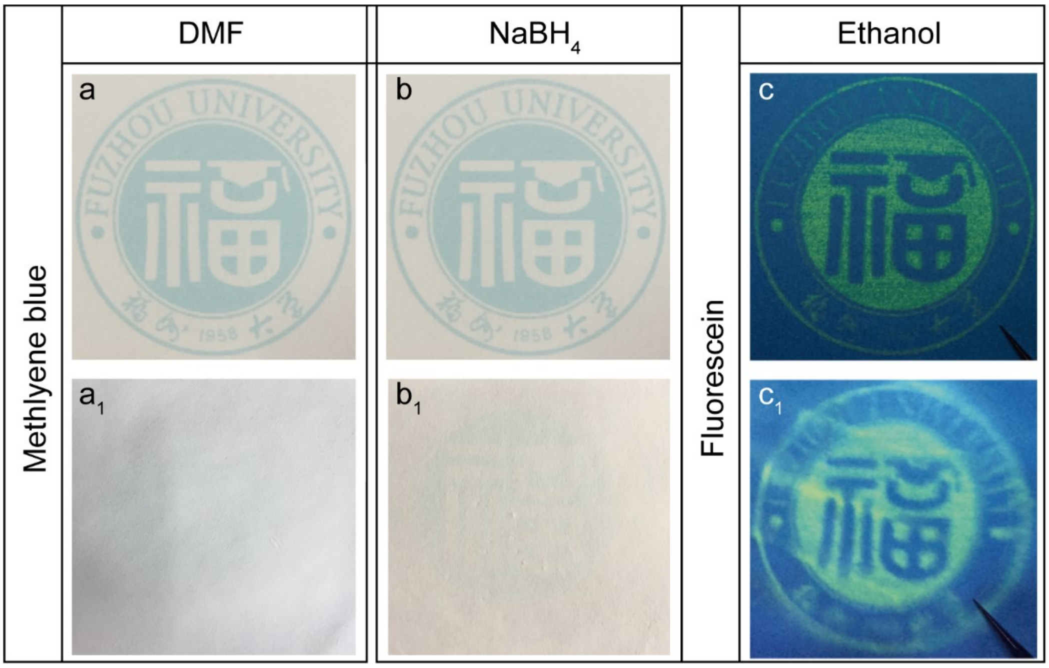

Fig. S3. The logo of Fuzhou university printed by methylene blue before (a) and after (a1) immersed in DMF under natural light; before (b) and after (b1) dealt with $NaBH_4$ under natural light. The logo printed by fluorescein dye before (c) and after (c1) immersed in ethanol under 365 nm UV light.

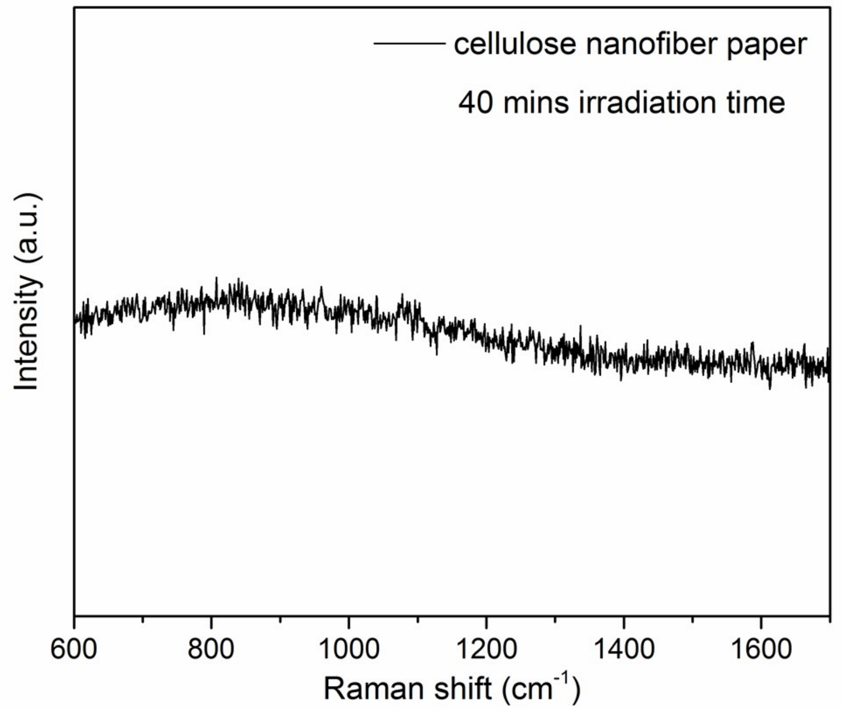

Fig. S4. The Raman spectrum of cellulose nanofiber paper after UV irradiation.
